# Assessing the Impact of an Integrated Community Care Program on Unplanned Hospital and Emergency Department Representations: Interrupted Time Series Analysis

**DOI:** 10.1111/jan.16808

**Published:** 2025-02-10

**Authors:** Brendan Shannon, Taya Collyer, Kelly‐Ann Bowles, Cylie Williams, Tanya Ravipati, Elise Deighton, Nadine E. Andrew

**Affiliations:** ^1^ Department of Paramedicine Monash University Melbourne Victoria Australia; ^2^ Peninsula Clinical School, Central Clinical School Monash University Melbourne Victoria Australia; ^3^ National Centre for Healthy Ageing Monash University and Peninsula Health Frankston Victoria Australia; ^4^ School of Primary and Allied Health Care Monash University Frankston Victoria Australia; ^5^ Community Care, Peninsula Health Frankston Victoria Australia

**Keywords:** community care, health services research, nursing home care

## Abstract

**Background:**

This study aimed to evaluate the impact of the Community Care Program, which was the amalgamation of three outreach services—post‐acute care, Residential In‐Reach, and the Hospital Admission Risk Program—into a single integrated care model. Specifically, we assessed its effects on unplanned hospital readmissions and emergency department re‐presentations at 30‐, 60‐ and 90‐days post‐enrolment.

**Methods:**

A pragmatic, real‐world, population‐based observational study was conducted using an interrupted time series analysis. The study included 4708 adult patients across two periods: pre‐amalgamation (November 2014–October 2016), and post‐amalgamation (May 2017–October 2018). Data were sourced from the National Centre for Healthy Ageing Data Platform, with statistical analyses conducted using Generalised Least Squares models to account for autocorrelation.

**Results:**

The study observed a significant increase in quarterly program enrolments post‐amalgamation, from 578 to 1011 per quarter. The 30‐day readmission rate decreased from 11.8% to 8.52% post‐amalgamation. However, interrupted time series analysis revealed no statistically significant changes in the slopes of readmission and emergency department re‐presentation rates after the program's amalgamation. The program did not result in significant changes in 60‐ or 90‐day outcomes.

**Conclusions:**

The amalgamation of post‐acute care, Residential In‐Reach, and the Hospital Admission Risk Program into the Community Care Program led to increased service utilisation without a significant impact on reducing unplanned hospital readmissions or emergency department re‐presentations. Although the program amalgamation demonstrated improved accessibility, its longer‐term impact remains inconclusive, highlighting the need for continuous refinement and further evaluation to optimise system efficiency. No patient or public contribution occurred in this study.

**Reporting Method:**

This study adhered to the STROBE guidelines for observational research.

**Summary**

- What problem did the study address?
  ○ The study addressed the impact of integrating multiple community outreach services on reducing unplanned hospital readmissions and ED presentations in patients with chronic and complex healthcare needs.
- What were the main findings?
  ○ The amalgamation increased program enrolments and decreased short‐term readmissions but did not show statistically significant long‐term effects on hospital or emergency department utilisation.
- Where and on whom will the research have an impact?
  ○ The research will impact healthcare policymakers and professionals involved in developing and implementing integrated community care models, particularly in settings managing patients with chronic and complex healthcare needs.
- What does this paper contribute to the wider global clinical community?
  ○ This study provides concrete evidence that while the integration of community‐based outreach services led to an immediate increase in patient enrolments, the overall trend throughout the study period was a steady decline in hospital readmissions and emergency department presentations. This is particularly relevant for health systems globally, as it suggests that program integration, even when it does not show immediate statistical significance in outcomes, can contribute to sustained improvements over time.
  ○ The results demonstrate that healthcare systems seeking to reduce acute care demand through integrated care models should consider long‐term trends rather than expecting immediate dramatic changes. The gradual decrease in readmissions and emergency department visits seen here can inform global efforts in chronic care management, showing that ongoing refinement and adaptation of services are crucial for success.
  ○ This paper highlights that integrated models like the one studied may benefit from further programmatic adjustments to maximise effectiveness. The findings encourage international health services to adopt a flexible, evolving approach to program integration, focusing on continuous improvement to achieve meaningful reductions in hospital admissions and emergency care usage over time.

## 1 Introduction

In contemporary healthcare systems, the challenge of managing patients with chronic and complex needs presents a difficult task (Osborn et al. 2015; Morphet et al. 2015). These patients, characterised by their frequent utilisation of emergency department (ED) visits and hospital admissions, pose a significant burden on resources and are often indicative of the broader issues within the healthcare continuum (Chang et al. 2023). The intricacies of their healthcare requirements demand a multifaceted approach that not only addresses their medical needs but also incorporates social support mechanisms to enhance the effectiveness of care provided (Fricke 2020). In response to these challenges, healthcare systems globally have been innovating and implementing various programs aimed at improving patient outcomes, reducing unnecessary hospital readmissions and optimising healthcare delivery, by intersecting care across the health and social continuum (Pang et al. 2021).

Among such initiatives, the Community Care Program, an initiative from the Victorian Department of Health, stands out as an approach in Melbourne, Australia. Situated within a broader health service, this program has brought together pre‐existing hospital based outreach services including post‐acute care (PAC) (Lim et al. 2003), Residential In‐Reach (RIR) (Rayner et al. 2017), and the Hospital Admission Risk Program (HARP) (Lawn et al. 2017) into a unified service (Shannon et al. 2023). In the Australian healthcare system, the HARP, PAC and RIR services represent targeted efforts to enhance patient care across different settings. HARP is designed to prevent unnecessary hospital admissions and readmissions by offering community‐based care and support to patients with chronic conditions, complex needs, or those at high risk of hospitalisation (Wan et al. 2021). This program focuses on integrated care that spans across the patient's journey, aiming to manage their conditions more effectively in the community setting. The PAC service on the other hand, facilitates the transition of patients from hospital to home by providing them with necessary support and rehabilitation services post‐discharge in the community (Hall et al. 2012). This promotes a smooth transition and may aid in the patient's recovery, reducing the likelihood of complications or readmissions. The RIR service specifically targets residents of aged care facilities, providing acute care interventions directly at the facility to avoid hospital admissions whenever possible (Kwa et al. 2021). Collectively, these services overlap in their overarching goal of optimising patient care through minimising unnecessary hospital admission and preventing ED presentations and ensuring that care is provided in the most appropriate setting.

This integration of these three distinct services into the single services of Community Care was a unique approach in the health service designed to provide a seamless, multidisciplinary outreach care model that supports patients regardless of specific program needs in the community. It was designed to provide a central access and referral triage point making it easier for patients, caregivers, and healthcare professionals to utilise and access the programs (Shannon et al. 2022). Despite the intuitive appeal of this amalgamation, empirical evidence on the long term impact on readmission rates remains inconclusive (Facchinetti et al. 2020). More specifically, the impact of integrating multiple outreach care services into a singular care model on hospital readmission rates and ED visits has yet to be thoroughly investigated. This gap in knowledge presents an opportunity to understand how integrated care models influence outcomes and healthcare utilisation patterns, which may lead to unintended consequences such as increased costs, higher hospital service use, and a decline in patient‐reported outcomes such as social support, capability and satisfaction with care (Ward et al. 2021).

Our study aimed to fill this gap by assessing the impact of the Community Care Program's services on re‐presentation rates to the ED, and unplanned hospital readmission rates at 30‐, 60‐ and 90‐days post‐enrolment in the program.

## 2 Methods

### 2.1 Design

A pragmatic, real‐world population‐based observational study was performed utilising retrospective analysis of collected administrative data within an interrupted time series (ITS) design (Bernal et al. 2017). Recognised as a robust quasi‐experimental method, ITS is particularly suited for evaluating intervention effects in non‐randomised contexts, especially in healthcare and public health settings (Bernal et al. 2017; Penfold and Zhang 2013). By analysing data across multiple time points before and after an intervention, ITS can identify underlying secular trends and account for potential autocorrelation. This makes it a valuable approach when concurrent control groups are impractical or unavailable, as was the case in our study.

### 2.2 Setting

The setting is Peninsula Health, a public health service in Victoria, Australia and the sole public health provider to the Frankston/Mornington Peninsula region, encompassing both urban and inner‐regional areas. Peninsula Health has an 850‐bed capacity and serves a community of 400,000 individuals across two acute hospitals, two subacute hospitals, and over 10 outpatient clinics and community health centres (Peninsula Health 2021).

### 2.3 Patient Selection

The cohort consisted of all adult patients enrolled in; (i) PAC, RIR and HARP in the pre‐amalgamation period (November 1, 2014, to October 31, 2016), and (ii) the Community Care program post‐amalgamation (May 1, 2017, to October 31, 2018). Overall eligibility for enrolment in the pre and post‐amalgamation services remained the same.

### 2.4 Study Periods

Three distinct time frames were analysed:

1. A historical ‘control’ period, henceforth referred to the pre‐amalgamation period, when the PAC, RIR and HARP programs were in place running individually from November 1, 2014, to October 31, 2016.
2. A 6‐month ‘washout’ or ‘transition’ period from November 1, 2016, to April 30, 2017, allowing for the assimilation of the amalgamated programs. This period accounted for the transitional phase during which workflows, systems, and processes were being established, making it unlikely for the full effects of the intervention to be observed. Incorporating a washout period aligns with standard practices in evaluating rollouts in real‐world healthcare settings using ITS designs (Mahant and Hall 2021).
3. The ‘intervention’ period henceforth referred to the post‐amalgamation period when the Community Care Program was fully established spanning from May 1, 2017, to October 31, 2018.

### 2.5 Intervention

The primary intervention was the amalgamation of PAC, RIR and HARP into the Community Care Program in November 2016. The consolidated program aimed to provide a more streamlined and efficient community care service, enhancing patient outcomes and reducing readmissions. The inception of the Community Care program was driven by the need for a cohesive, multidisciplinary outreach care model (Shannon et al. 2023). The overarching goal of the Community Care program is to offer a spectrum of services, from medical consultations to nursing and specialised health services to patient population groups at risk of readmissions and high healthcare resource utilisation. Notably, both the pre‐ and post‐amalgamation care models did not adhere to rigid criteria for patient enrolment. Instead, its flexible approach welcomes referrals from multiple sources. Acute care clinicians in the ED, Paramedics and general practitioners (GPs) often recommend the program to aid patient discharge processes or provide a transition of care. Similarly, staff from residential aged care homes can refer individuals. Additionally, the program is open to direct patient referrals or those made by their caregivers, whether formal or informal. This enhanced capacity to transition care across disciplines without the need for a formal referral enables timely and effective care, ensuring that patients receive the necessary support promptly and efficiently.

### 2.6 Data Sources

This research leveraged data sourced from the National Centre for Healthy Ageing (NCHA) Healthy Ageing Data Platform (Andrew et al. 2023). A Monash University Peninsula Health partnership, the NCHA provides a comprehensive data repository through the integration, internal linkage and curation of data across the entire public health service, including all inpatient and outpatient or community‐based services. It offers insights into the health profiles of around 1 million individuals spanning a decade. For the purposes of this study, specific variables were selected from three distinct datasets within the NCHA Data Platform for the required time periods, as detailed in Table S1. The datasets provided comprehensive patient‐level data, capturing diagnostic codes, length of stay, ED visits, hospital admission data and Community Care services data.

### 2.7 Co‐Variate Definitions and Data Management

Data for our study was sourced from the secure NCHA data platform, with all records having been de‐identified for privacy. Demographic variables encompassed factors such as age, gender, marital status, need for an interpreter, regular living arrangements and socioeconomic background. Socioeconomic status was determined using the Index of Relative Socioeconomic Advantage Disadvantage (IRSAD), a measure provided by the Australian Bureau of Statistics (Australian Bureau of Statistics 2011). This index is derived from national census data, focusing on aspects such as education level, occupation type, household conditions, and income, all associated with specific state suburb codes. A higher score on the IRSAD indicates a reduced level of socioeconomic disadvantage.

For our clinical variables we identified comorbidities based on the Elixhauser comorbidity index, which utilised specific International Statistical Classification of Diseases and Related Health Problems, Tenth Revision, Australian Modification (ICD‐10‐AM) code sets (Riley et al. 2024). To ensure comprehensive coding of comorbidities, we incorporated all primary and secondary ICD‐10‐AM codes linked to hospital admissions and ED presentations during the study duration and the preceding 5 years. These codes were subsequently categorised per the Elixhauser comorbidity index guidelines (Sharma et al. 2021). The Elixhauser comorbidity index was chosen due to its widespread use in administrative datasets, its extensive coverage of conditions, and its superior predictive power for readmission and in‐hospital mortality risks compared to Charleson comorbidity index (Kim and Bae 2023). See Table S2 for ICD‐10 codes used in the study. Unplanned hospital presentations were based on unplanned ED only return visits and hospital readmissions during the study timeframe. Planned and statistical hospital admissions were removed by excluding all hospital admission episodes with an admission type of planned admission, statistical readmission, admission from waiting list, other planned admission, or statistical separation. Patients under 18 years of age, as well as presentations related to dialysis care, antineoplastic chemotherapy and immunotherapy, were excluded from the study to ensure the focus remained on unplanned presentations. These exclusions were identified using ICD‐10‐AM. For a detailed list of the specific ICD‐10‐AM codes used in the study, please refer to Table S3. To distinguish between ED‐only visits and subsequent admissions, any ED presentation that culminated in a hospital stay was categorised as a singular admission. Only those ED visits without an associated hospital admission were classified exclusively as ED only presentations.

### 2.8 Statistical Analyses

Baseline characteristics and outcomes were compared across the study periods using chi‐squared tests for categorical variables, *t*‐tests for means, and Kruskall–Wallis tests for medians. The effects of the program amalgamation on readmission rates were investigated via ITS analysis. This method provides a robust mechanism to ascertain the effects of interventions in real‐world settings by controlling for pre‐existing trends, especially when a system‐wide change has occurred and randomisation is not feasible (Bernal et al. 2017). The analysis included examining the monthly proportion of patients who returned to the hospital with unplanned ED only visits or had unplanned readmissions to the hospital within 30, 60, and 90 days. This approach enabled us to monitor changes over time and assess the impact of the program amalgamation on these rates. Changes in the level and slope (trend over time) between the pre‐amalgamation and post‐amalgamation periods were compared, excluding the 6‐month transition period (i.e., the interruption period).

In implementing the ITS analysis, we developed time series regression models. These models were structured to include terms for time, serving the purpose of capturing underlying trends within the data. Additionally, an indicator variable for the post‐amalgamation period was integrated to identify immediate level changes due to the program amalgamation. Furthermore, to discern the post‐amalgamation trend alterations, an interaction term between time and the post‐amalgamation indicator was incorporated. We chose monthly epochs for this analysis to balance the need for data granularity with statistical robustness. Monthly data provides a detailed view of trends and seasonality which can be accounted for in autoregressive integrated moving average (ARIMA) models (Schaffer et al. 2021), which may be obscured in yearly analyses and overly volatile in weekly datasets. Furthermore, monthly re‐presentation and readmission rates are often used in Australian healthcare services as a key performance indicator to be reported on (Radley et al. 2016; Garrubba et al. 2016). Due to the monthly nature of the time series data and seasonality likely to be present, autocorrelation was assessed. The initial phase of this assessment employed the Durbin‐Watson statistic as a diagnostic tool to detect the presence of autocorrelation. This was followed by a more detailed examination using Autocorrelation Function (ACF) and Partial Autocorrelation Function (PACF) plots. These diagnostics informed subsequent model choices, leading to the application of Generalised Least Squares (GLS) models. Depending on the detected autocorrelation patterns, these models were adjusted to incorporate autoregressive (AR), moving average (MA), or combined (ARMA) structures in order to correct for autocorrelation. Further detail on model selection can be found in Appendix S1. We considered *P* values of < 0.05 as statistically significant result. All statistical analyses were carried out using R software. See Appendix S2 for reporting against the STROBE Checklist.

## 3 Ethics

This study was granted ethical approval by the Human Research Ethics Committee (HREC) at Peninsula Health service (HREC/LNR/18/PH/35) and Monash University (MUHREC 18799).

## 4 Results

In this study, a total of 4708 patients were included, with 1026 patients evaluated during the 24 monthly epochs of the pre‐amalgamation period, and 3682 patients evaluated over the 18 monthly epochs of the post‐amalgamation period. There was a significant increase in the mean number of quarterly enrolments into the program/s from the pre‐amalgamation to the post‐amalgamation period, with 578 enrolments per quarter (SD, 52.5) in the pre‐amalgamation period compared to 1011 (SD, 383) in the post‐amalgamation period (*p* = 0.002). Patient demographics varied between the pre‐amalgamation and post‐amalgamation groups. Specifically, post‐amalgamation patients were older (60 years vs. 67 years), had a lower percentage of males (47.9% vs. 43.1%), and fewer were married or in de facto relationships (69.6% vs. 61.9%) (Table 1).

**TABLE 1:** Demographics table.

Patient characteristic	Pre‐amalgamation	Post‐amalgamation	*p*
Enrolments per quarter in program/s, mean (SD)	**578 (52.5)**	**1011 (383)**	**0.002**
Total population with ED or hospital admission	**1026**	**3682**	
Gender (Male), *n* (%)	**491 (47.9)**	**1586 (43.1)**	**0.007**
Age, mean (SD)	**60.51 (19.46)**	**66.72 (16.80)**	**< 0.001**
Married/de facto, *n* (%)	**713 (69.6)**	**2275 (61.9)**	**< 0.001**
Interpreter required, *n* (%)	7 (0.7)	50 (1.4)	0.112
Usual accommodation, *n* (%)			
Other	72 (7.0)	221 (6.0)	0.169
Private residence	826 (80.5)	3057 (83.0)	
Residential aged care home	128 (12.5)	404 (11.0)	
Socioeconomic position, *n* (%)*			
Most disadvantaged	162 (15.9)	592 (16.1)	0.152
Second most disadvantaged	168 (16.5)	579 (15.7)	
Third most disadvantaged	434 (42.6)	1454 (39.5)	
Fourth most disadvantaged	181 (17.8)	715 (19.4)	
Least disadvantaged	74 (7.3)	338 (9.2)	

*Note:* Bold values indicate statistically significant results for the peer reviewers.

* Derived using the index of relative social advantage and disadvantage.

Abbreviations: ED, emergency department; SD, standard deviation.

Statistically significant differences in the health profiles of enrolled patients between the pre and post‐amalgamation periods were noted (Table 2). Notably, there were significant reductions in the proportion of patients with congestive heart failure (25.2% pre‐ to 19.2% post‐amalgamation) and chronic pulmonary disease (27.8% pre‐ to 19.1% post‐amalgamation). Similarly, significant decreases were observed in conditions such as fluid and electrolyte disorders (43.9% pre‐ to 37.1% post‐amalgamation) and unhealthy alcohol use (20.0% pre‐ to 10.0% post‐amalgamation). The overall index of comorbidity decreased in the post‐amalgamation period with a median score of 6 compared to 9 in the pre‐amalgamation period.

**TABLE 2:** Clinical characteristics table.

Patient characteristic, *n* (%)	Pre‐amalgamation	Post‐amalgamation	*p*
Congestive heart failure	**259 (25.2)**	**707 (19.2)**	**< 0.001**
Cardiac arrhythmias	**322 (31.4)**	**1024 (27.8)**	**0.028**
Valvular disease	26 (2.5)	101 (2.7)	0.798
Pulmonary circulation disorders	**88 (8.6)**	**234 (6.4)**	**0.015**
Peripheral vascular disorders	68 (6.6)	252 (6.8)	0.862
Hypertension (uncomplicated)	**274 (26.7)**	**866 (23.5)**	**0.039**
Hypertension (complicated)	7 (0.7)	36 (1.0)	0.488
Paralysis	72 (7.0)	235 (6.4)	0.511
Other neurological disorders	125 (12.2)	396 (10.8)	0.217
Chronic pulmonary disease	**285 (27.8)**	**704 (19.1)**	**< 0.001**
Diabetes (uncomplicated)	51 (5.0)	234 (6.4)	0.116
Diabetes (complicated)	222 (21.6)	781 (21.2)	0.801
Hypothyroidism	21 (2.0)	68 (1.8)	0.775
Renal failure	**199 (19.4)**	**569 (15.5)**	**0.003**
Liver disease	**162 (15.8)**	**294 (8.0)**	**< 0.001**
Peptic ulcer disease	6 (0.6)	26 (0.7)	0.839
AIDS/HIV	0 (0.0)	2 (0.1)	1.000
Lymphoma	9 (0.9)	61 (1.7)	0.093
Metastatic cancer	57 (5.6)	209 (5.7)	0.943
Solid tumour without metastasis	44 (4.3)	201 (5.5)	0.158
Rheumatoid arthritis	18 (1.8)	97 (2.6)	0.133
Coagulopathy	**127 (12.4)**	**256 (7.0)**	**< 0.001**
Obesity	42 (4.1)	136 (3.7)	0.616
Weight loss	**244 (23.8)**	**690 (18.7)**	**< 0.001**
Fluid and electrolyte disorders	**450 (43.9)**	**1367 (37.1)**	**< 0.001**
Blood loss anaemia	37 (3.6)	112 (3.0)	0.417
Deficiency anaemia	102 (9.9)	416 (11.3)	0.241
Unhealthy alcohol use	**205 (20.0)**	**370 (10.0)**	**< 0.001**
Substance use disorder (excluding alcohol)	**127 (12.4)**	**188 (5.1)**	**< 0.001**
Psychoses	**48 (4.7)**	**106 (2.9)**	**0.006**
Depression	**206 (20.1)**	**430 (11.7)**	**< 0.001**
Elixhauser Comorbidity Index scores, median [IQR]	**9.00 [0.00, 21.00]**	**6.00 [0.00, 16.00]**	**< 0.001**

*Note:* Hypertension (complicated) = with chronic complications such as hypertensive heart disease or hypertensive renal disease; Diabetes (complicated) = with chronic complications such as diabetic retinopathy, neuropathy or nephropathy. Bold values indicate statistically significant results for the peer reviewers.

Abbreviation: IQR, inter quartile range.

Table 3 provides the detailed specifics on readmission rates and ED‐only re‐presentations before and after the program's amalgamation. Across the study periods, the most notable changes were observed in the 30‐day readmission and ED representation rates. The proportion of patients with 30‐day readmission rate decreased from 11.8% at the start of the pre‐amalgamation period to 8.52% by the end of the post‐amalgamation period. Similarly, the 30‐day ED only re‐presentation rate saw a drop from 7.10% to 2.15%.

**TABLE 3:** Rates of Readmission and Emergency Department only representations before and after program implementation.

Variable	Start of pre‐amalgamation period^a^ (%) [95% CI]	End of pre‐amalgamation period^a^ (%) [95% CI]	Start of post‐amalgamation period^a^ (%) [95% CI]	End of post‐amalgamation period^a^ (%) [95% CI]
Readmission				
30‐day readmission	11.8 [11.2, 12.4]	11.7 [10.7, 12.6]	11.6 [9.72, 13.52]	8.52 [7.21, 9.84]
60‐day readmission	15.1 [14.5, 15.7]	13.7 [13.16, 14.30]	13.5 [12.49, 14.46]	12.2 [11.06, 13.29]
90‐day readmission	16.7 [16.0, 17.3]	14.8 [14.22, 15.44]	14.8 [13.61, 16.04]	13.3 [12.02, 14.55]
ED only representation				
30‐day ED representation	7.10 [6.75, 7.46]	4.38 [4.04, 4.72]	4.65 [4.13, 5.18]	2.15 [1.53, 2.78]
60‐day ED representation	8.34 [7.8, 8.8]	4.96 [4.50, 5.45]	5.25 [4.53, 5.97]	2.20 [1.35, 3.05]
90‐day ED representation	9.21 [8.74, 9.69]	5.66 [5.20, 6.12]	5.72 [4.93, 6.51]	2.78 [1.92, 3.64]

^a^ Estimated from the interrupted time series model.

**FIGURE 1:**
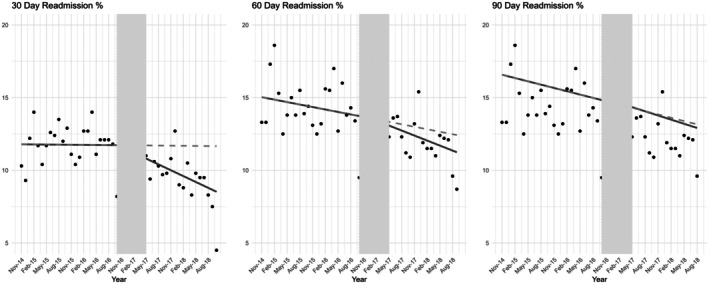
Effect of the community care program's amalgamation on 30‐, 60‐ and 90‐day hospital readmission rates: Interrupted time series analysis. Individual points depict the proportion of patients readmitted at each of the specified intervals in month periods. The shaded area highlights the transition phase during which the community care program was integrated. Solid lines depict regression models for the pre‐and post‐amalgamation periods, offering insights into the trends before and after the program's implementation. Dashed lines indicate the projected readmission rates in the post‐amalgamation period, grounded on the observed trends from the control period.

**FIGURE 2:**
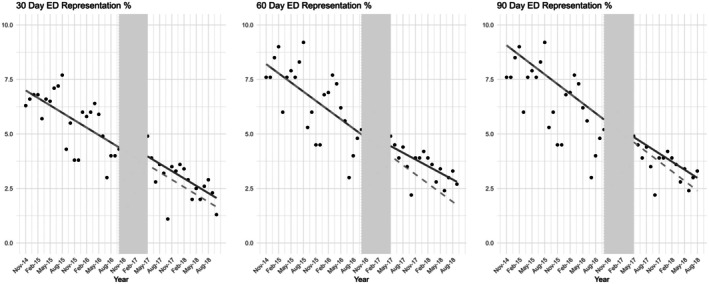
Effect of the community care program's amalgamation on 30‐, 60‐ and 90‐day ED only re‐presentation rates: Interrupted time series analysis. Individual points depict the proportion of patients readmitted at each of the specified intervals in month periods. The shaded area highlights the transition phase during which the community care program was integrated. Solid lines depict regression models for the pre‐ and post‐amalgamation periods, offering insights into the trends before and after the program's implementation. Dashed lines indicate the projected readmission rates in the post‐amalgamation period, grounded on the observed trends from the control period.

The results of the interrupted time series analysis are visually depicted in Figures 1 and 2, highlighting the trends before and after the program's amalgamation.

Table 4 delineates the effects of the community care program's amalgamation on all‐cause readmission rates and ED‐only representations. For hospital admissions, the 30‐day readmission rate showed a non‐significant trend towards reductions in readmissions post‐amalgamation, with a change in slope of −0.132 (95% CI: −0.281 to 0.02, *p* = 0.0819). The 60‐day and 90‐day readmission rates remained much the same post‐amalgamation, with slope changes of −0.06 (95% CI: −0.24 to 0.15, *p* = 0.66) and 0.00 (95% CI: −0.21 to 0.22, *p* = 0.98), respectively. The ED only re‐presentation rates at 30‐, 60‐, and 90‐days post‐discharge also remained largely unchanged following the program integration, with slope changes of 0.01 (95% CI: −0.08 to 0.13, *p* = 0.64), 0.02 (95% CI: −0.07 to 0.19, *p* = 0.391), and 0.01 (95% CI: −0.11 to 0.17, *p* = 0.6593), respectively. Comparing predicted outcomes to the counterfactual, the overall effect of the amalgamation showed reductions of −2.89, −1.82 and −0.68 for 30‐day, 60‐day, and 90‐day readmissions. For ED‐only re‐presentations, changes were −0.27, +0.41 and −0.33 at 30, 60, and 90 days.

**TABLE 4:** Interrupted time series analysis—monthly change in slope and level of time trends.

Variable	Change in slope^a^ (post vs. pre) [95% CI]	*p*	Change in level post‐amalgamation^b^ [95% CI]	*p*	Overall effect of amalgamation^c^ (counterfactual vs. predicted)
Readmission					
30‐day readmission	−0.132 [−0.281, 0.02]	0.08	−0.765 [−1.7, 0.18]	0.432	−2.89
60‐day readmission	−0.06 [−0.24, 0.15]	0.66	−0.20 [−1.45, 1.06]	0.76	−1.82
90‐day readmission	0.00 [−0.21, 0.22]	0.98	0.07 [−1.37, 1.51]	0.928	−0.68
ED only representation					
30‐day ED representation	0.01 [−0.08, 0.13]	0.64	0.39 [−0.30, 1.07]	0.28	−0.27
60‐day ED representation	0.02 [−0.07, 0.19]	0.391	0.41 [−0.52, 1.35]	0.39	0.41
90‐day ED representation	0.01 [−0.11, 0.17]	0.659	0.21 [−0.76, 1.17]	0.68	−0.33

^a^ Slopes represent the estimated average change per month.

^b^ Level represents the immediate change on amalgamation implementation.

^c^ Indicates whether readmission or re‐presentation rates differed from the counterfactual estimate based on time period trends.

## 5 Discussion

The amalgamation of PAC, RIR, and HARP into a single Community Care program represents a shift in Peninsula Health service system's approach to addressing the complex needs of patients. Although statistically significant reductions in hospital readmission rates and ED‐only representations at 30‐, 60‐, and 90‐day were not achieved, non‐significant reductions were observed in the context of a doubling of enrolments per quarter and minimal increase in resourcing. The observed trend aligns with the objectives of integrated care frameworks, which focus on seamless care transitions and enhanced follow‐up care with the ultimate aim of reducing the need for acute care services post‐discharge readmissions and ED presentations.

As the average length of time enrolled in the program has been reported to be 50 days (Shannon et al. 2023), the absence of significant findings in 60‐day and 90‐day readmission rates, along with ED‐only representations, shows ambiguity regarding the long‐term effectiveness of the program in preventing readmissions particularly once people are discharged from the program/s. This brings into focus the necessity for a continual refinement of the program's structure and focus of activities. Supporting evidence from other studies reinforces this perspective. For instance, a study analysing the restructuring of residential in reach services (Kwa et al. 2021) demonstrated that after the RIR service was restructured to involve more geriatric expertise and improved continuity of care, there was a 67% increase in RIR activity along with an 11% reduction in the rate of ED presentations and a significant reduction in associated costs, even with an increase in patient enrolment. This suggests that well‐structured programs with specific enhancements in care coordination and expertise can lead to improved patient outcomes and system efficiency. Furthermore, research by Dai et al. (Dai et al. 2021) on transitioning from a subacute to an acute geriatric outreach service, led by a geriatrician team, demonstrated a significant decrease in monthly hospital admissions from residential aged care facilities and reduced overall risk of admission by 36.1%. These findings exemplify the substantial benefits of employing acute, targeted interventions over subacute strategies, particularly for patients in residential care. Our study's findings, in conjunction with these supportive examples from the literature, reinforce the notion that with more precise refinements and targeted implementation strategies, the Community Care program could manifest more substantial benefits. This emphasises the potential in reducing short‐term readmissions, when optimally structured and executed, highlighting the need for ongoing evaluation and adaptation of the program to maximise its effectiveness in meeting the needs of patients with complex needs.

An interesting result of the amalgamation of the program in this study was the significant increase in program enrolments from the pre‐amalgamation to the post‐amalgamation period. This increase in utilisation might reflect greater awareness and accessibility of the services, suggesting that the amalgamation did address the assumed under‐utilisation of the program previously noted in qualitative studies investigating the health professional experience of the program (Shannon et al. 2022). Previous literature has shown that the amalgamation of pre‐existing healthcare services is a complex process (O'Toole et al. 2002), that when done correctly can lead to improved financial stability and patient care (Hoelscher and Sprick 1999). However, literature has noted that the focus should be on integrating various medical sectors to enhance coordination and efficiency, rather than simply redistributing services (Botezat et al. 2013) which was focus of the amalgamation seen in this study. The near doubling of enrolments in the post‐amalgamation period shows an increased and sustained need within the health service for this program. This increase in utilisation reflects a positive response to the amalgamation of services, suggesting that the integration efforts were successful in addressing perceptions of previous under‐utilisations (Shannon et al. 2022).

However, this increase in enrolments must be viewed in light of the demographic and clinical changes observed between the pre‐amalgamation and post‐amalgamation periods. These changes, including an older average age and variations in the prevalence of certain comorbidities, might have influenced the outcomes of the program. Older patients and those with specific health conditions might require more intensive or different types of care strategies, which could affect the program's effectiveness in reducing readmissions. Despite this the patients enrolled in the program had a predominance of characteristics putting them at higher risk of readmission including lack of a spouse (Sharmin et al. 2021; Hasan et al. 2010), advanced age (Amarasingham et al. 2015), the presence of multiple chronic conditions (Low et al. 2016) and longer hospital stays, all known to be associated with higher 30‐day readmission rates (Donze et al. 2013). The observed decrease in overall readmissions during the post‐amalgamation period, juxtaposed with a lower comorbidity index but increased average age among patients, raises several considerations. Firstly, it suggests that the amalgamation of services within the Community Care program may have benefited specific demographic groups differently. It is possible that the integration led to more targeted or effective care for older individuals, some of whom may have had lower overall comorbidity levels. The expansion in numbers post‐amalgamation may also have meant that people with less severe conditions were able to enter the program/s, promoting a shift towards proactive care for a larger, yet less acutely ill cohort, highlighting an effective expansion of the program's scope. To gain deeper insights into these dynamics, additional qualitative studies exploring patient experiences could uncover operational realities and areas for improvement. Furthermore, economic evaluations are warranted, as similar community‐based programs have shown promising cost‐effectiveness compared to control groups, suggesting a potentially beneficial economic impact of the Community Care Program (Hernandez et al. 2024).

## 6 Limitations

This study's observational design, which leverages administrative data from a specific geographic and healthcare setting in Melbourne, inherently limits its ability to establish causality and may not capture detailed clinical nuances affecting patient outcomes. Additionally, the generalisability of findings is restricted due to geographic specificity. However, the geographic location contains socioeconomic and geographic diversity enhancing its applicability to most scenarios. The primary outcome measure of re‐presentations and readmissions does not adequately assess quality of life or other health outcomes that may be considered meaningful to the patients involved. The study time frame (November 2014–October 2018) also presents challenges, as patterns of disease incidence and healthcare utilisation can shift over time. Although the study included 24 monthly epochs before the intervention, above the 8 epochs recommended for ITS (Bernal et al. 2017), this timeframe reflects the maximum feasible pre‐intervention window, given the program's November 2016 start date and our access to reliable administrative data dating back to November 2014. While more baseline observations could theoretically detect smaller effect sizes, the incremental benefit diminishes after establishing a stable trend, and extending further back may introduce inconsistencies due to changes in administrative coding practices in our study setting.

Finally, we used an ITS design without a concurrent analysis in a formal control group due to the uniqueness of the program amalgamation which was implemented service wide. Hence, no suitable comparison site existed. While non‐equivalent control group designs can be used in such cases, no feasible control site existed for this study. In these scenarios such as our study, numerous methodological papers recognise ITS as a rigorous approach that can capture underlying trends (Bernal et al. 2017; Penfold and Zhang 2013), providing greater insights than a simple pre‐post comparison. While a concurrent control would have strengthened causal inference by ruling out time‐varying external factors (Lopez Bernal et al. 2018), the absence of such a control is not uncommon in ITS analyses involving large‐scale real‐world interventions. Nevertheless, the non‐controlled ITS approach does adjust for non‐time varying confounders and is an appropriate and robust method for evaluating the program's impact within the context of this study.

## 7 Conclusion

The amalgamated Community Care program led to a significant increase in enrolments, but importantly this did not result in an overall increase in unplanned hospital readmissions or ED re‐presentations. The significant increase in program enrolments post‐amalgamation indicates improved accessibility and utilisation of the program. The implications of these findings suggest that integrated care models like the Community Care Program have the potential to improve patient outcomes by providing more accessible and coordinated care. However, the inconclusive long‐term impacts highlight the need for continuous refinement and adaptation of such programs to sustain their effectiveness and the need for ongoing long‐term monitoring of effectiveness. Further investigation, including qualitative assessments and economic evaluations, is necessary to fully understand the program's impact on healthcare system outcomes and to identify areas for improvement. These insights can inform future efforts to develop and implement similar integrated care models, ultimately aiming to enhance the efficiency and effectiveness of healthcare delivery and reduce the burden on acute care services.

## Author Contributions

Study concept and design: B.S., N.E.A., K.‐A.B., C.W. Acquisition of the data: T.R., N.E.A., B.S. Analysis and interpretation of the data: B.S., T.C., N.E.A., E.D. Drafting of the manuscript: B.S., N.E.A., T.C. Critical revision of the manuscript for important intellectual content: B.S., T.C., K.‐A.B., C.W., T.R., E.D., N.E.A. Statistical expertise: T.C., B.S., N.E.A.

## Conflicts of Interest

The authors declare no conflicts of interest.

## Peer Review

The peer review history for this article is available at https://www.webofscience.com/api/gateway/wos/peer‐review/10.1111/jan.16808.

## Supporting information


Data S1.

## Data Availability

No data are available. Due to ethical and legal limitations, sharing patient‐level data from this study is not permissible. However, the corresponding author can provide aggregated data outputs and coding that underpin the study's conclusions upon reasonable request, contingent on obtaining approval from the relevant data custodians.
