# Supplementary material for: Assessing the Impact of an Integrated Community Care Program on Unplanned Hospital and Emergency Department Representations: Interrupted Time Series Analysis
Source: J Adv Nurs. 2025 Feb 10;81(10):6578–88. doi: 10.1111/jan.16808 (PMC12460973; doi:10.1111/jan.16808)
Supplement: Supplementary file 1 — Data S1. [file JAN-81-6578-s001.docx]

**Supplementary files**

**Table S1.** Variables included in data extraction

| Dataset | Demographic variables | Clinical Variables | Healthcare utilization variables |
| --- | --- | --- | --- |
| Community care program variable dataset | Birth Month |  | Presented to ED |
|  | Birth Year |  | Had Hospital Admission |
|  | Gender |  | Referral Received Date |
|  | Marital Status |  | Episode End Date |
|  | Postcode |  | Contact Date |
|  | Needs Interpreter |  |  |
|  | Living Arrangement |  |  |
|  | Carer Availability |  |  |
|  | Usual Accommodation |  |  |
|  | Date of Death |  |  |
| ED variables dataset | Birth Month | Injury Cause | Arrival Date Time |
|  | Birth Year | Main Injury | Arrival Mode |
|  | Gender | Human Intent | Visit Type |
|  | Marital Status | Triage Category | Triage Date Time |
|  | Postcode |  | Departure Destination |
|  | Needs Interpreter |  | Departure Date |
|  | Living Arrangement |  | Expected Discharge Date |
| Admission variables dataset | Admission Date | Admission Type | Discharge Date |
|  | Birth Month | Admission Specialty | Length of Stay |
|  | Birth Year | Admission Care Type | Discharge Method |
|  | Gender | Died during Episode |  |
|  | Marital Status | Date of Death |  |
|  | Postcode | DRG Code |  |
|  | Needs Interpreter | ICU Stay |  |
|  | Living Arrangement | Primary Diagnosis (ICD10-AM) |  |
|  |  | Secondary Diagnosis (ICD10-AM) |  |

**Table S2.** The ICD- 10 AM codes used in comorbidity package to classify the Elixhauser comorbidity indices

| Elixhauser comorbidity index | ICD-10 codes |
| --- | --- |
| Congestive heart failure | I09.9, I11.0, I13.0, I13.2, I25.5, I42.0, I42.5 - I42.9, I43.x, I50.x, P29.0 |
| Cardiac arrhythmias | I44.1 - I44.3, I45.6, I45.9, I47.x - I49.x, R00.0, R00.1, R00.8, T82.1, Z45.0, Z95.0 |
| Valvular disease | A52.0, I05.x - I08.x, I09.1, I09.8, I34.x - I39.x, Q23.0 - Q23.3, Z95.2 - Z95.4 |
| Pulmonary circulation disorders | I26.x, I27.x, I28.0, I28.8, I28.9 |
| Peripheral vascular disorders | I70.x, I71.x, I73.1, I73.8, I73.9, I77.1, I79.0, I79.2, K55.1, K55.8, K55.9, Z95.8, Z95.9 |
| Hypertension (uncomplicated) | I10.x |
| Hypertension (complicated) | I11.x - I13.x, I15.x |
| Paralysis | G04.1, G11.4, G80.1, G80.2, G81.x, G82.x, G83.0 - G83.4, G83.9 |
| Other neurological disorders | G10.x - G13.x, G20.x - G22.x, G25.4, G25.5, G31.2, G31.8, G31.9, G32.x, G35.x - G37.x, G40.x, G41.x, G93.1, G93.4, R47.0, R56.x |
| Chronic pulmonary disease | I27.8, I27.9, J40.x - J47.x, J60.x - J67.x, J68.4, J70.1, J70.3 |
| Diabetes, uncomplicated | E10.0, E10.1, E10.9, E11.0, E11.1, E11.9, E12.0, E12.1, E12.9, E13.0, E13.1, E13.9, E14.0, E14.1, E14.9 |
| Diabetes, complicated | E10.2 - E10.8, E11.2 - E11.8, E12.2 - E12.8, E13.2 - E13.8, E14.2 - E14.8 |
| Hypothyroidism | E00.x - E03.x, E89.0 |
| Renal failure | I12.0, I13.1, N18.x, N19.x, N25.0, Z49.0 - Z49.2, Z94.0, Z99.2 |
| Liver disease | B18.x, I85.x, I86.4, I98.2, K70.x, K71.1, K71.3 - K71.5, K71.7, K72.x - K74.x, K76.0, K76.2 - K76.9, Z94.4 |
| Peptic ulcer disease, excluding bleeding | K25.7, K25.9, K26.7, K26.9, K27.7, K27.9, K28.7, K28.9 |
| AIDS/HIV | B20.x - B22.x, B24.x |
| Lymphoma | C81.x - C85.x, C88.x, C96.x, C90.0, C90.2 |
| Metastatic cancer | C77.x - C80.x |
| Solid tumour without metastasis | C00.x - C26.x, C30.x - C34.x, C37.x - C41.x, C43.x, C45.x - C58.x, C60.x - C76.x, C97.x |
| Rheumatoid arthritis/collagen vascular diseases | L94.0, L94.1, L94.3, M05.x, M06.x, M08.x, M12.0, M12.3, M30.x, M31.0 - M31.3, M32.x - M35.x, M45.x, M46.1, M46.8, M46. |
| Coagulopathy | D65 - D68.x, D69.1, D69.3 - D69.6 |
| Obesity | E66.x |
| Weight loss | E40.x - E46.x, R63.4, R64 |
| Fluid and electrolyte disorders | E22.2, E86.x, E87.x |
| Blood loss anaemia | D50.0 |
| Deficiency anaemia | D50.8, D50.9, D51.x - D53.x |
| Alcohol abuse | F10, E52, G62.1, I42.6, K29.2, K70.0, K70.3, K70.9, T51.x, Z50.2, Z71.4, Z72.1 |
| Drug abuse | F11.x - F16.x, F18.x, F19.x, Z71.5, Z72.2 |
| Psychoses | F20.x, F22.x - F25.x, F28.x, F29.x, F30.2, F31.2, F31.5 |
| Depression | F20.4, F31.3 - F31.5, F32.x, F33.x, F34.1, F41.2, F43.2 |

.x means all the sub-codes from the main ICD-10 code

**Table S3.** The ICD-10 AM codes used to exclude planned hospital admissions

|  | ICD-10 codes |
| --- | --- |
| Care involving dialysis | Z490, Z491, Z492, Z992, Z998 |
| Care related to antineoplastic chemotherapy and immunotherapy | Z510, Z511, Z5112, Z08, Z79899, |

**Supplementary Appendix S1. Model equation and selection**

**Model Equation**

**T**he statistical model used in our analysis is as follows:

Y_t_ = β_0_ + β_1_(Time_t_) + β_2_(Intervention_t)_ + β_3_(Time_t_ × Intervention_t_) + ε_t_

Where:

- Y_t_: Outcome variable (30-, 60-, 90-day readmission or ED representation rate) at time *t*.
- Time_t_: Continuous variable representing the time period (both pre- and post-intervention).
- Intervention_t_: Binary indicator for pre- (0) or post-intervention (1) period.
- Time_t_ × Intervention_t_: Interaction term to estimate the slope change post-intervention.
- ε_t_: Error term, adjusted for autocorrelation using Generalized Least Squares (GLS) modelling.

This model captures:

1. Baseline trends (β_1_): The trajectory of the outcome variable before the intervention.
2. Immediate level change (β_2_): The immediate effect of the intervention on the outcome.
3. Change in slope (β_3_): The long-term effect of the intervention, reflected in the rate of change post-intervention compared to the pre-intervention period.

**Dependent and independent variables**

Dependent variables were the monthly rates of unplanned hospital readmissions and emergency department-only re-presentations at 30-, 60-, and 90-days post-enrolment.

Independent variables included time (continuous), the intervention indicator (binary), and their interaction.

**Model Selection**

Given the temporal structure of our data, an assessment of autocorrelation was required to ensure the validity of our findings. The Durbin-Watson statistic served as our primary diagnostic tool for detecting autocorrelation's presence. A Durbin-Watson statistic significantly different from 2 indicates autocorrelation; values approaching 0 suggest positive autocorrelation, while values closer to 4 indicate negative autocorrelation. However, the Durbin-Watson test alone is not always sufficient for determining the specific nature or degree of autocorrelation present in the data. For further analysis, we employed Autocorrelation Function (ACF) and Partial Autocorrelation Function (PACF) plots. ACF plots provide a visual representation of the correlation between observations in a time series separated by various time lags. In contrast, PACF plots display the partial correlation of an observation with its lag, controlling for the correlations of the observations at all shorter lags. The patterns observed in these plots guided our selection of appropriate statistical models to account for autocorrelation. In cases where autocorrelation was detected, we used Generalized Least Squares (GLS) models. The flexibility of GLS models lies in their ability to accommodate various forms of autocorrelation through the specification of different correlation structures such as:

- Autoregressive [AR] models were considered when the PACF plot showed a sharp cut-off while the ACF plot displayed a more gradual decline. This indicated that the value of a time series at a particular time point is a linear combination of its previous values. The order of the AR model (e.g., AR(1), AR(2)) was determined based on the lag at which the PACF plot cut off.
- Moving Average [MA] models were applied in situations where the ACF plot exhibited a sharp cut-off, and the PACF displayed a more gradual decrease. This suggested that the current value of the series is a linear combination of its past errors. The order of the MA model was chosen based on the ACF plot's cut-off point.
- Autoregressive Moving Average [ARMA] models combined the AR and MA components to model series with autocorrelation patterns not adequately captured by pure AR or MA models alone. The selection of ARMA models was guided by the iterative process of model fitting and checking, where both ACF and PACF plots were used to refine the model's order.

The selection process for the optimal model involved evaluating the model fit using criteria such as the Akaike Information Criterion (AIC) and the Bayesian Information Criterion (BIC), alongside diagnostic checks for residual autocorrelation. This iterative process ensured that the final model adequately corrected for autocorrelation, allowing for true estimates. All statistical analyses, including the autocorrelation assessment, model fitting, and diagnostic checks, were conducted in R software (Version 4.3.1). R's suite of packages for time series analysis (forecast, nlme, tseries) provided the necessary tools for this modelling process.

**Supplementary Appendix S2. STROBE Checklist**

|  | Item No | Recommendation | Page No |
| --- | --- | --- | --- |
| **Title and abstract** | 1 | (*a*) Indicate the study’s design with a commonly used term in the title or the abstract | 1 |
|  |  | (*b*) Provide in the abstract an informative and balanced summary of what was done and what was found | 1 |
| Introduction | | | |
| Background/rationale | 2 | Explain the scientific background and rationale for the investigation being reported | 3 |
| Objectives | 3 | State specific objectives, including any prespecified hypotheses | 5 |
| Methods | | | |
| Study design | 4 | Present key elements of study design early in the paper | 5 |
| Setting | 5 | Describe the setting, locations, and relevant dates, including periods of recruitment, exposure, follow-up, and data collection | 5 |
| Participants | 6 | (*a*) Give the eligibility criteria, and the sources and methods of selection of participants. Describe methods of follow-up | 5 |
|  |  | (*b*) For matched studies, give matching criteria and number of exposed and unexposed | N/A |
| Variables | 7 | Clearly define all outcomes, exposures, predictors, potential confounders, and effect modifiers. Give diagnostic criteria, if applicable | 6- 9 |
| Data sources/ measurement | 8* | For each variable of interest, give sources of data and details of methods of assessment (measurement). Describe comparability of assessment methods if there is more than one group | 8-9 |
| Bias | 9 | Describe any efforts to address potential sources of bias | 9 |
| Study size | 10 | Explain how the study size was arrived at | 9 |
| Quantitative variables | 11 | Explain how quantitative variables were handled in the analyses. If applicable, describe which groupings were chosen and why | 6-10 |
| Statistical methods | 12 | (*a*) Describe all statistical methods, including those used to control for confounding | 9-10 |
|  |  | (*b*) Describe any methods used to examine subgroups and interactions | N/A |
|  |  | (*c*) Explain how missing data were addressed | 9-10 |
|  |  | (*d*) If applicable, explain how loss to follow-up was addressed | N/A |
|  |  | (*e*) Describe any sensitivity analyses | N/A |
| Results | | |  |
| Participants | 13* | (a) Report numbers of individuals at each stage of study—eg numbers potentially eligible, examined for eligibility, confirmed eligible, included in the study, completing follow-up, and analysed | 11 |
|  |  | (b) Give reasons for non-participation at each stage | N/A |
|  |  | (c) Consider use of a flow diagram | N/A |
| Descriptive data | 14* | (a) Give characteristics of study participants (eg demographic, clinical, social) and information on exposures and potential confounders | 11 |
|  |  | (b) Indicate number of participants with missing data for each variable of interest | N/A |
|  |  | (c) Summarise follow-up time (eg, average and total amount) | N/A |
| Outcome data | 15* | Report numbers of outcome events or summary measures over time | 14 |
| Main results | 16 | (*a*) Give unadjusted estimates and, if applicable, confounder-adjusted estimates and their precision (eg, 95% confidence interval). Make clear which confounders were adjusted for and why they were included | 14 |
|  |  | (*b*) Report category boundaries when continuous variables were categorized | N/A |
|  |  | (*c*) If relevant, consider translating estimates of relative risk into absolute risk for a meaningful time period | N/A |
| Other analyses | 17 | Report other analyses done—eg analyses of subgroups and interactions, and sensitivity analyses | 16 |
| Discussion | | | |
| Key results | 18 | Summarise key results with reference to study objectives | 17 |
| Limitations | 19 | Discuss limitations of the study, taking into account sources of potential bias or imprecision. Discuss both direction and magnitude of any potential bias | 20 |
| Interpretation | 20 | Give a cautious overall interpretation of results considering objectives, limitations, multiplicity of analyses, results from similar studies, and other relevant evidence | 17-20 |
| Generalisability | 21 | Discuss the generalisability (external validity) of the study results | 19 |
| Other information | | | |
| Funding | 22 | Give the source of funding and the role of the funders for the present study and, if applicable, for the original study on which the present article is based | N/A |
